# Increasing STEM career interest: The role of out-of-school time STEM programs designed for underrepresented minorities

**DOI:** 10.1371/journal.pone.0336418

**Published:** 2025-11-07

**Authors:** Maria Dresser, Kelly Miller, Gerhard Sonnert, Philip Sadler

**Affiliations:** 1 Physics Department, Harvard University, Cambridge, Massachusetts, United States of America; 2 School of Engineering and Applied Sciences, Harvard University, Cambridge, Massachusetts, United States of America; 3 Harvard-Smithsonian Center for Astrophysics, Harvard University, Cambridge, Massachusetts, United States of America; California State University Chico, UNITED STATES OF AMERICA

## Abstract

The creation of a large and diverse STEM workforce is a national imperative in the U.S. Despite significant efforts to improve equitable STEM educational and hiring practices, disparate employment in STEM fields across racial and ethnic demographics persists. Educational researchers and practitioners have increasingly focused on out-of-school time STEM programs as a potential avenue for boosting high school students’ interest in pursuing STEM careers. However, many studies on the efficacy of such programs rely on data from single programs with small sample sizes. The present work uses our nationally representative sample of 14,176 U.S. college students to investigate the relationship between out-of-school time STEM program attendance and students’ reported STEM career interests. Our analysis shows that students who, during their high school years, attended an out-of-school time STEM program designed specifically for underrepresented minority students had 2.4 times the odds of reporting an interest in a STEM career at the end of high school, compared to those who did not attend any out-of-school time STEM program (p<0.001). By contrast, students who attended a general population STEM program (not specifically designed for underrepresented minority students) had only 1.3 times the odds of expressing an interest in a STEM career at the end of high school, compared to those who did not attend any out-of-school time STEM program (p<0.001). Additionally, those who attended an underrepresented minority STEM program had 1.9 times the odds of aspiring to a STEM career, compared to those who attended a general population program (p<0.001). This is the first study to use nationally representative data to compare underrepresented minority focused and general out-of-school time STEM programs. Given these promising results, this work encourages further development and funding of out-of-school time STEM programs designed for underrepresented minorities to foster a diverse and equitable STEM workforce.

## 1. Introduction

Advancements in Science, Technology, Engineering, and Mathematics (STEM) fields are at the forefront of tackling contemporary global issues such as climate change, food security, public health, and clean infrastructure development. STEM occupations in the U.S. are growing at twice the rate of other occupations [1], and population projections suggest that the demographic makeup of the U.S. will contain a majority of people of color by 2050 [2]. Yet, disparities in STEM employment based on racial and ethnic demographics remain. For example, while currently one third of the U.S. population identifies as Black or Hispanic, only 14% of the STEM workforce falls into these demographic categories [3–5].

This underrepresentation can, to a substantial degree, be attributed to systemic barriers inhibiting student persistence, interest, or necessary preparation to pursue STEM. One study found that in California schools with high minority populations, 16% of mathematics teachers were under-qualified, while in schools with majority White populations, only 4% of mathematics teachers were under-qualified [6]. The hiring of under-qualified teachers is in part due to limited funding. In a study of 6 major metropolitan regions across the U.S., school districts with a majority of Black and Hispanic students were allocated 40% less money per student on average, as compared to neighboring school districts with a majority of White students [7]. Exposure to mathematics and science courses is a significant predictor for choosing a STEM major in postsecondary education [8], yet a nationally representative sample showed that Black and Hispanic students are statistically significantly less likely to be placed in advanced mathematics courses in the 9^th^ grade, compared to White students [9].

Under-qualified teachers, limited funding, and inequitable course enrollment practices contribute to disparities in formal STEM learning experiences for underrepresented minority (URM) students. Efforts to advance equity in STEM education typically focus on either school-based reforms or out-of-school time (OST) STEM programs that expand access beyond the classroom.

### 1.1 School-based reforms

Improved assessment. Scholars have highlighted the importance of culturally sustaining assessment—classroom practices that recognize learning as socially and culturally situated rather than neutral. Traditional assessments often privilege the experiences of White and middle-class students, while framing other cultural backgrounds as deficits [10]. Culturally sustaining approaches instead value diverse forms of sense-making and emphasize student agency, autonomy, and recognition [11]. For example, self and peer grading can allow students to reflect on their own progress toward learning goals, thereby fostering responsibility and ownership of their learning. Additionally, two-stage exams, in which students first complete an exam individually and then retake it collaboratively in groups, have been shown to reduce test anxiety, build peer connections, and narrow equity gaps in STEM course outcomes [12].

Culturally relevant content. Another strand of work emphasizes designing course projects that resonate with students’ lived experiences and community concerns. For instance, in one chemistry course, the instructor structured several learning units to focus on the chemical processes at the heart of the water crisis in Flint, Michigan. Such content can be particularly meaningful for students of color, as they demonstrate the real-world utility of STEM knowledge and promote a sense of empowerment [13].

Curricular and pedagogical reforms. Active learning approaches—including think–pair–share, small-group work, and real-time polling—have consistently been shown to improve learning outcomes and reduce disparities across racial and ethnic groups. A large meta-analysis of 41 STEM courses found that active learning reduced equity gaps in exam performance between URM and majority students by approximately one-third, compared to traditional lecture-based courses [14]. Some schools have adopted a “looping” model, in which students remain with the same teacher across multiple years. Research indicates that looping can build stronger relationships and continuity, leading to greater gains in mathematics competencies compared to students taught by different teachers each year [15].

While these in-school practices are effective, as aforementioned, limited funding to schools with high minority populations can inhibit teachers from having the resources and proper training to implement changes in their classroom such as these. This has led to an increased interest in out-of-school time STEM programs and their contribution to shrinking the gap in STEM participation [16–21].

### 1.2 Out-of-school time STEM programs

Out-of-school time (OST) STEM programs can take many different forms, such as summer camps, residential research internships, competitions, and clubs. Some programs have been specifically developed for students considered to be URMs in STEM. Various components unique to, or emphasized in, URM STEM programs have received attention in the literature, ranging from interactions with same race role models, mentors, and peers [22–29], to explicit discussions of underrepresentation and use of culturally relevant pedagogy [27,29–32]. Participation opportunities for parents [22,29,33] and financial incentives [26,28,31,34] have also been noted in the literature on URM STEM programs.

This literature indicates that participation in URM STEM programs is associated with improved academic performance, a greater sense of readiness for STEM education, and enhanced academic skills [26–28,31,33,34]. Studies have shown that comparatively many students who attended URM STEM programs go on to attend college, pursue STEM majors, and complete their degrees [24,26,28,31,32,34]. Participating in a URM STEM program has also been found to result in improvements in STEM attitudes, self-assurance, and self-efficacy [24,26,27,29,33–36]. In several studies, participants reported developing a sense of belonging and forming a meaningful academic community within STEM [23,27,30,31]. Below, we report in detail on several URM STEM programs to provide relevant context on the components of and benefits of such programs previously documented in the literature.

The Wright STEPP program was established in 1988 as an annual 5-week STEM summer program for students in 7^th^-10^th^ grade [22]. The program recruited 160 students primarily from city public schools, the majority of whom were first-generation college or economically disadvantaged, with a significant portion identifying as ethnic or racial minorities [31,37]. The program included STEM coursework in a variety of fields, STEM career exposure, role model exposure, and workshops on written and oral communication. Additionally, students and their parents were provided with information about scholarships, financial aid, college housing, and college placement testing. The Wright STEPP program provided a full ride scholarship to Wright State University for any student who completed the 4-year summer program with a 3.5 GPA [31,37].

Two former participants of Wright STEPP, Leroy L. Long III and Bobby L. Elam, reported on their participation in the program [31]. One of their comments highlighted the impact a same race mentor can have: “I never dreamed of becoming a college professor, but I knew it was possible after meeting Black men like Dr. Beck and Dr. Ruby Mawasha. Their leadership roles with Wright STEPP helped me understand the power and impact of earning the academic title of ‘Dr.’” [31]. According to the report, having Black mentors who were aware of the marginalization of Black people in STEM fields, yet believed in their capabilities, was a meaningful experience [31]. They additionally noted that developing a community of Black peers who were like minded and wanted to excel in STEM was beneficial. The authors indicated that attending this program increased their STEM performance in high school, increased STEM course taking in college, increased chances of majoring in STEM, and increased their chances of completing a STEM degree.

Hernández-Matías et al. (2023) reported on the experiences of 127 students who participated in Seeds of Success, a STEM program for Hispanic girls designed to foster connections with culturally similar role models and inspire STEM career interest [29]. The program took place in an out of school online format with interactive workshops, hands on learning kits, community-based STEM projects, and a coding/robotics workshop. Students interacted with mentors who identified as Hispanic women in small group and one-on-one meetings. Mentors received explicit guidance on encouraging a growth mindset, normalizing the experience of setbacks, and sharing components of their identity that the students could identify with. Additionally, mentors were trained in cultural awareness and sensitivity skills. The program included culturally relevant STEM Ambassador projects, where the Hispanic students designed and developed community-focused STEM projects. From pre- and post-surveys, it was found that students reported increased understanding of STEM careers and STEM opportunities, positive experiences with role models, increased STEM self-efficacy, STEM identity, and STEM attitudes [29].

The Loma Linda University summer research internship in biomedical science was an 8-week paid research internship program serving a high school student population of 132 students, of which 81% identified as racially or ethnically URM and 69% identified as female [26]. Students were paired with ethnically diverse mentors to spend 40 hours per week on a hands-on lab-based research project. In addition to participating in this research, students were given career exposure and taught literature review skills, responsible research conduct, and presentation skills. Attendees reported gains in research skills, research self-efficacy, and confidence in their scientific ability and scientific skills. Students also reported a 64-percentage point increase in interest in scientific research-based careers. Follow up data from Loma Linda showed that 49% of program attendees were in college with 67% majoring in STEM and 55% enrolling in graduate programs [26].

The Research Internship and Science Education (RISE) program was a 2-year research internship in biomedicine designed for high school students, beginning in the summer of their sophomore year and concluding after the summer following their senior year [34]. Recruits for this program were drawn from public schools in Atlanta, with 87% of the participants self-identifying as Black. These 39 students completed research projects, instruction in college level biology, SAT preparation, college application support, and career exposure. Students were paired with URM mentors whenever possible. The internship program provided stipends of $2,500 over the summer and $1,000 over the academic year to the participating high school students. The stipends were intended to offset hours the students may have needed to work. Surveys from the RISE program showed that afterwards, 76% of students felt convinced a science career was right for them, and 81% felt better prepared and more motivated to pursue STEM careers. RISE program students scored higher on the SAT and maintained higher GPAs than their peers at the same school, on average. Ninety-seven percent of RISE participants attended 4-year institutions, 61% majored in STEM, and 20% conducted research [34].

The above programs show promising results. However, these results and others reported in the literature [23–25,28,30,33,35] are limited to small sample sizes of localized programs. In this study, we expand upon the previous literature by answering the research question: “Is URM STEM program attendance associated with increased STEM career interest?”

## 2. Theoretical framework

This study draws on Social Cognitive Career Theory (SCCT) and Critical Race Theory (CRT) to understand the benefits of, and need for, URM STEM programs. Together, these frameworks highlight both the individual-level processes that shape career development and the systemic inequities that shape educational experiences for underrepresented students.

SCCT explains how individuals develop career interests, make choices, and persist in educational and occupational settings [38]. It emphasizes the interaction of cognitive-person factors (e.g., self-efficacy, outcome expectations, and goals), background factors (e.g., race, gender, culture), and environmental factors (e.g., social support, educational climate, and economic conditions) [38,39]. Self-efficacy, in particular, is shaped by mastery experiences, vicarious learning, social persuasion, and affective states [40]. URM STEM programs foster these conditions through hands-on lab activities, mentorship, and peer support, which help students build confidence and persistence in STEM.

While SCCT focuses on individual career development, CRT highlights the systemic barriers that create the need for URM-specific programs. CRT argues that racism is deeply embedded in educational systems and that curricula often reflect dominant cultural norms, reproducing inequities [41]. From this perspective, URM students’ challenges are not simply individual or resource-based but structural, rooted in environments that systematically marginalize their identities and limit opportunities. URM STEM programs counter these barriers by incorporating culturally relevant curricula, engaging mentors who are culturally aware, and, when possible, providing role models who reflect students’ racial and ethnic backgrounds.

## 3. Data and methods

### 3.1 Survey and sample

We developed the survey for the study “Collaborative Research: A Study of How Pre-College Informal Activities Influence Female Participation in STEM Careers.” This study was funded by the National Science Foundation and carried out at the Science Education Department of the Harvard-Smithsonian Center for Astrophysics. Some portions of the survey were adapted from several co-authors’ prior survey work [42]. We administered the survey via professors to college students across the U.S. who were enrolled in mandatory first-year English classes during the fall of 2017. The choice of mandatory English classes was deliberate, ensuring that students from a wide range of academic disciplines and majors were included in the sample. The survey included 33 items to assess student’s career plan development, early school science and mathematics experiences, high school background, STEM-related interests, student characteristics, and family characteristics (see the supporting PDF for a view of all items on the survey). To obtain responses from a diverse and representative sample of college students, we used a stratified random sampling method. Stratification was informed by the NCES database and was based on the nature of the institution, whether it was a 2-year or 4-year college, as well as the size of the student population, categorized as small (N = 1,000−7,713), medium (N = 7,800−21,482), and large (N = 21,786−133,211). Recruitment occurred between February to October of 2017, and data were collected from August 2017 to December 2017. The result was a nationally representative sample of college students, consisting of responses from 15,725 students of 592 instructors at 119 institutions. The sample size reported in this study used for logistic regression results includes only 14,176 students due to some missingness which persisted after multiple imputations were performed (reported in the missing data section below).

We collected survey reliability and validity evidence through a test-retest study involving 137 undergraduate students at a large U.S. university. Participants were drawn from mandatory first-year English courses taken by all students at the institution, ensuring that the pilot sample reflected the diversity of the intended target population—college students in the U.S. Reliability coefficients were determined for item blocks, ranging from 0.4 to 0.9. An average reliability of 0.7 for continuous items was established through Pearson correlations, a value that is considered acceptable [43]. For non-continuous items, an average reliability of 0.5 was established through Spearman correlations and found to be acceptable [44]. Additional content related validity evidence was collected through expert evaluations from a group of science educators and science education researchers.

Sample characteristics were averaged across the imputed datasets, as shown in Table 1. In our sample, 23% of students identified as Hispanic, 10% identified as Black, 13% identified as Asian or Pacific Islander, 46% identified as White, and 7% identified as Native American, Alaskan Native, multiracial, or “other”. One percent of the data on race and ethnicity remained missing after multiple imputations. Self-identified female students accounted for 54% of the sample, and non-female students accounted for 46% of the sample. First-generation college students made up 45% of the sample, while non-First-generation students represented 53% of the sample. Information on first generation status remained missing for 2% of the sample.

**Table 1:** Participant Demographics. Students were marked as Hispanic regardless of race chosen and were not marked in any other category to prevent double counting. Some groups may not sum to 100% due to rounding.

Race/Ethnicity	N	%
Hispanic	3,674	23
Black	1,571	10
Asian or Pacific Islander	1,984	13
White	7,162	46
Native American, Alaskan Native, multiracial, other	1,127	7
Missing	206	1
**Gender**		
Female	8,512	54
Non-female	7,213	46
**First Generation College Status**		
First Generation	7,037	45
Not First Generation	8,346	53
Missing	342	2

This study was approved by the Harvard University-Area Committee on the Use of Human Subjects. It was determined this study met the criteria for exemption per the regulations found at 45 CFR 46.101(b)(2). The students were informed about the research on the cover page of the questionnaire and were told that participation was optional. If the students filled out the questionnaire, this was taken as implicit consent. Personally identifiable information was not collected; thus, all data analysis was anonymized. Only students 18 years or older were allowed to participate.

### 3.2 Handling of missing data

Missing data were handled with multiple imputation via chained equations in R using the mice package [45]. It was determined that 50% of cases were complete, thus, 50 imputations were performed in accordance with suggestions in the literature [46]. Dichotomous variables were imputed with logistic regression modelling, while continuous variables were imputed via predictive mean matching. Auxiliary variables were chosen via the four-step selection method suggested by van Buuren and Groothuis-Oudshoorn using the quickpred function in mice [45,46]. The correlation was set to 0.2, a cutoff used to choose only a subset of auxiliary variables based on their correlation with our variables of interest [46]. The minpuc argument was set to 0.3, eliminating predictors for imputations with less than 30% of usable cases. Thus, we did not include all variables in our dataset during imputations. Including all variables is generally discouraged and would have been computationally expensive with 600 variables in our data set. Our multiple imputation procedure resulted in a small amount of persistent missing data (Table 1). All variables were checked for plausibility of the imputations, and no issues were uncovered.

### 3.3 Dependent variable

Given our interest in STEM career aspirations, the primary dependent variable was students’ report of their career interests at the end of high school. For our analysis, we categorized careers including mathematics, physical sciences, life sciences, engineering, and computer sciences as STEM careers. All other careers were categorized as non-STEM (see Table 2). This categorization resulted in a dichotomous variable (0: interest in non-STEM career at end of high school; 1: interest in STEM career at end of high school). The designation of STEM vs. non-STEM was determined based on the U.S. Department of Education’s categorization [47].

**Table 2:** Careers presented on the survey. These careers were presented without any designation of STEM or non-STEM. Careers were classified into these categories during data processing.

STEM Careers
Astronomer
Biologist
Chemist
Earth/Environmental scientist
Physicist
Other scientist
Engineer
Computer scientist/Programmer/IT Specialist
Mathematician/Statistician
STEM teacher
**Non-STEM Careers**
Medical doctor (e.g., physician, dentist, vet.)
Health professional (e.g., social worker, nurse, pharmacist)
Other teacher
Anthropologist/Archaeologist
Social scientist (e.g., psychologist, sociologist)
Humanities professional (e.g., historian, language specialist, writer, philosopher)
Visual artist (e.g., painter, sculptor, architect, computer artist/animator)
Performing artist (e.g., actor, musician, dancer)
Business person (e.g., entrepreneur, manager)
Lawyer
Politician
Athlete/Coach
Military personnel
Other non-STEM related career

### 3.4 Primary independent variables

To answer our research question, we analyzed information provided by participants on their attendance of STEM programs. Participants reported engaging in various types of STEM programs, including STEM clubs, summer camps, internships, and job shadowing (see Table 3). Additionally, participants were asked if they had attended any STEM programs designed specifically for underrepresented minorities (URMs). To separate the impact of general population STEM programs not specifically designed for URMs from those that were, a three-tier variable was coded. Students who did not choose any options from the STEM program list and did not report attending a URM STEM program were coded as 0 for “no program.” Students who chose at least one option from the STEM program list but did not report attending a URM STEM program were coded as 1 for “general population STEM program.” All remaining students who reported that they attended a STEM program designed for URMs were coded as 2 for “URM STEM program.”

**Table 3:** STEM programs listed in the survey.

STEM Programs
STEM-related extracurricular clubs/teams at school
STEM-related clubs/teams outside of school
Group organization (e.g., Girl Scouts, Boy Scouts, 4H)
Maker/DIY STEM activities/events
Overnight STEM programs (at museums, science centers etc.)
STEM Cafes (eat, drink, chat about STEM with professionals)
STEM-related vacation or summer camps
STEM-related programs that collect/analyze data for scientists (e.g., citizen science)
STEM-related lectures or talks (online or in person)
STEM-related courses/workshops outside of school (online or in person)
STEM-related leadership conferences
Science fairs
Robotics competitions
Engineering competitions
Computing/IT competitions
STEM-related academic/research summer programs
STEM-related career days
Tours of STEM-related settings (e.g., hospital, vet’s office, lab)
STEM-related job-shadowing
STEM-related internships
Work/Volunteer in a STEM-related setting (e.g., hospital, vet’s office, lab, camp, museum, zoo)

### 3.5 Control variables

To obtain a clearer view of the relationship between STEM program participation and STEM career aspirations at the end of high school, we controlled for baseline interest in a STEM career at the beginning of high school. Interest in a STEM career at the end of high school can be influenced by many factors, beyond the initial interest and STEM program participation. Consequently, we incorporated several other control variables in our statistical models, related to student demographics, background, and preparation. An overview of control variables is included in Table 4. Prior mathematics performance was controlled for, based on students’ reported mathematics SAT score. If a student provided an ACT score, it was converted to an SAT score. For ease of interpretation, mathematics SAT scores were standardized with a mean of zero and standard deviation of one. Race and ethnicity were controlled for and coded as Hispanic, Black, Asian, White, and Other. Students who identified as Hispanic were coded as such regardless of race chosen, thus all other racial categories do not include Hispanic students. Students coded as “Other” include those who marked they were Native American or Alaskan Native and those who marked multiple races or “other” on the survey. The collapsing of these into our “Other” code was done to address limited sample size for those groups. Gender was controlled for in a dichotomous variable “Female” (0: not female; 1: female). Students were able to choose “other” as a gender. Due to extremely low counts (N = 114 or 0.7%), “other” was included in the “not female” category rather than creating a variable with 3 levels. First-generation college status was accounted for based on the student’s report of their parent’s education level (0: not first-generation; 1: first-generation). The operationalization of this variable followed the Higher Education Act, in which a student is considered a first-generation college student if neither parent received a bachelor’s degree. Other demographic variables that would typically be of use to those interested in diversity and inclusion in STEM, such as disability status, sexual orientation, LGBTQ identity, and income were unfortunately not available to us in our current dataset.

**Table 4:** Overview of logistic regression variables.

Variable	Description
Control Variables	
Race/ethnicity	0 = White non-Hispanic; 1 = Black non-Hispanic; 2 = Hispanic; 3 = Asian or Pacific Islander non-Hispanic; 4 = Other
Female	0 = not Female; 1 = Female
STEM Career interest at beginning of high school	0 = Uninterested in a STEM career at beginning of H.S.; 1 = Interested in a STEM career at beginning of H.S.
Math SAT/ACT	Standardized continuous variable for Math SAT scores. If a student provided an ACT score, it was converted to an SAT score.
First-Generation college student	0=At least one parent received a bachelor’s degree or higher; 1=Neither parent received a bachelor’s degree
Primary Independent Variable	
STEM program	0 = Did not attend any STEM program; 1 = Attended a general population STEM program and said no to attending a URM program; 2 = Attended a URM STEM program
Dependent Variable	
STEM career interest at end of high school	0 = Uninterested in a STEM career at end of H.S.; 1 = Interested in a STEM career at end of H.S.

### 3.6 Analysis

Six logistic regression models predicting STEM career interest at the end of high school were estimated in the statistical software program R version 4.2.3 [48]. In model 1, only control variables were included to develop an understanding of the relationship between controls and end of high school STEM career interest. To address our research question, model 2 included all control variables and the three-tier variable related to STEM programs. We also estimated models with interactions (all combinations between Race/ethnicity, Gender, STEM Program, and First-Generation College Student, with all controls in place). These interaction models are included in supplementary information S2-S4 Tables. Given the large size of the data set, we chose to set alpha to 0.001 for interactions. None of the interactions were significant. For non-interaction terms, alpha was set to 0.05. In models 1, 2, S2, S3, S4, the specified categorical variable’s reference was the category designated with the code “0” in Table 4. In model S1, the STEM program variable was set with the reference “general population STEM program” to allow for a direct comparison between a URM STEM program and a general population STEM program. After logistic regression models were built, average adjusted predicted probabilities were computed for model 2 with the marginaleffect package version 0.15.1 in R [49]. Plots were generated with ggplot2 version 3.5.1 [50].

## 4. Results

### 4.1 Descriptive statistics

Table 5 presents the participation rates of students in STEM programs, organized by demographic categories. For STEM programs designed for underrepresented minorities, Black students had the highest participation rate at 10%. Next, 6% of Hispanic students, 6% of Asian students, 5% of “other” students, and 2% of White students reported attending a URM program. Black students also had the highest proportion of non-participation, with 48% reporting they did not attend any STEM program. This is followed by 46% of Hispanic students, 45% of “other” students, 43% of White students, and 34% of Asian students reporting they did not attend any STEM program. Those reporting they attended a general population STEM program include 60% percent of Asian students, 55% of White students, 50% of “other” students, 48% of Hispanic students, and 42% of Black students. Table 5 also shows attendance of program type separated by gender and first-generation status.

**Table 5:** Percent of students that attended each type of STEM program or no program, broken up by demographic groups. Rows may not sum to 100% due to rounding.

Race/Ethnicity	Attended URM STEM program (%)	Attended general population STEM program (%)	Did not attend any STEM program (%)
Hispanic (N = 3,674)	6	48	46
Black (N = 1,571)	10	42	48
Asian or Pacific Islander (N = 1,984)	6	60	34
White (N = 7,162)	2	55	43
Native American, Alaskan Native, multiracial, other (N = 1,127)	5	50	45
**Gender**			
Female (N = 8,512)	5	53	42
Non-female (N = 7,213)	3	50	46
**First Generation College Status**			
First Generation (N = 7,037)	4	49	47
Not First Generation (N = 8,346)	4	55	41

### 4.2 Logistic regression models

In all models, as seen in Tables 6 and S1-S4, beginning of high school STEM career interest was associated with a large increase in the odds of end of high school STEM career interest, compared to students without a beginning of high school interest (p<0.001). Similarly, in all models, females were significantly less likely to have an interest in a STEM career at the end of high school, compared to non-females (p<0.001). Math SAT scores were also a strong predictor of end of high school STEM interest (p<0.001).

**Table 6:** Logistic regression results from multiply imputed data predicting STEM career interest at the end of high school. Model 1 includes control variables only. Model 2 adds STEM program attendance as the primary predictor variable. Shaded cells are statistically significant at the 0.05 level or lower.

	Model 1: Controls only			Model 2: Controls and Predictors		
Variables	Odds Ratio (95% CI)	Standard Error	P-value	Odds Ratio (95% CI)	Standard Error	P-value
Intercept	0.27 (0.24, 0.29)	0.05	<0.001	0.23 (0.21, 0.26)	0.06	<0.001
Controls						
Beginning of H.S. STEM Career Interest	8.21 (7.49, 9.00)	0.05	<0.001	8.02 (7.31, 8.80)	0.05	<0.001
Black	0.88 (0.74, 1.04)	0.09	0.14	0.83 (0.69, 0.98)	0.09	0.03
Hispanic	0.98 (0.86, 1.10)	0.06	0.69	0.95 (0.84, 1.07)	0.06	0.37
Asian	1.29 (1.13, 1.48)	0.07	<0.001	1.24 (1.08, 1.43)	0.07	0.002
Other race/eth	1.04 (0.87, 1.24)	0.09	0.68	1.02 (0.86, 1.22)	0.09	0.80
Female	0.56 (0.51, 0.61)	0.05	<0.001	0.54 (0.49, 0.59)	0.05	<0.001
First Generation	0.98 (0.89, 1.08)	0.05	0.68	0.99 (0.90, 1.10)	0.05	0.88
Math SAT	1.35 (1.28, 1.43)	0.03	<0.001	1.32 (1.25, 1.40)	0.03	<0.001
Predictors						
General population STEM program vs. No program				1.31 (1.19, 1.43)	0.05	<0.001
URM STEM program vs. No program				2.41 (1.94,3.01)	0.11	<0.001
Other model measures	AIC	BIC	N	AIC	BIC	N
	14046	14114	14176	13964	14047	14176

From model 2 (Table 6), we see that attending a general population STEM program or attending a URM STEM program were both positive predictors of STEM career interest. Students who attended a URM STEM program had a 2.4 times increase in the odds of having a STEM career interest at the end of high school, compared to those who did not attend any program (p<0.001). This contrasts with only a 1.3 times increase in the odds for a student who attended a general population STEM program, compared to a student who did not attend any program (p<0.001). Additionally, in model 3 (S1 Table) students who attended a URM program had 1.9 times the odds of expressing STEM career interest, compared to those who attended a general population program (p<0.001). Models 4–6 in Tables S2-S4 show that interactions between program type and race, ethnicity, gender, and first-generation status are non-significant, with a large odds ratio still observed for URM STEM programs. This indicates URM STEM programs were effective for all students who attended regardless of their demographic background.

### 4.3 Predicted probabilities

**Fig 1:**
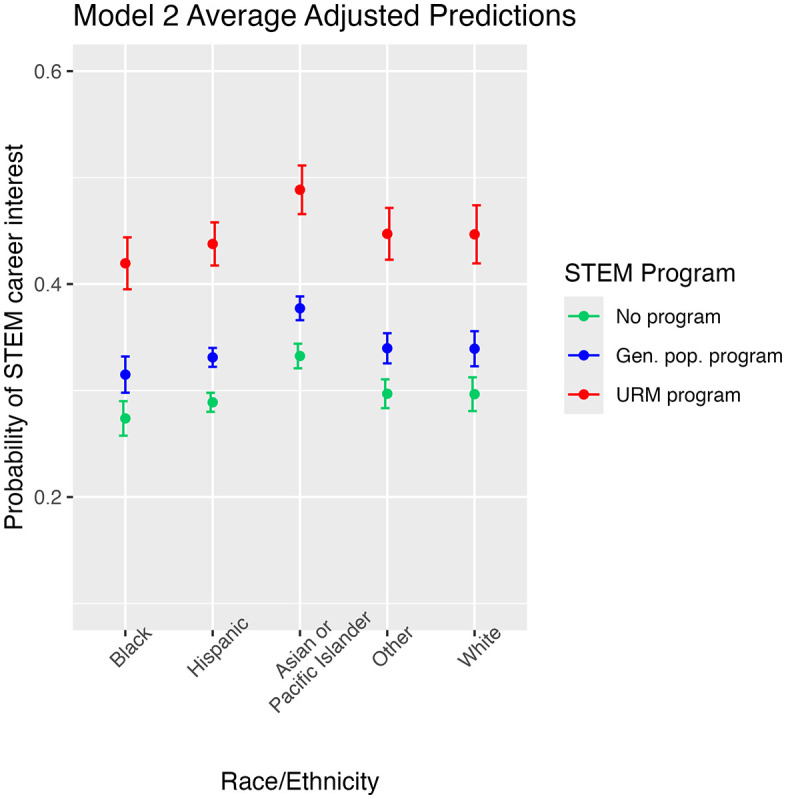
Average adjusted predicted probability of STEM career interest at the end of high school from model 2. Error bars represent one standard error of the predicted mean. Gen. pop. program: general population STEM program not specifically designed for underrepresented minorities. URM Program: STEM program designed specifically for underrepresented minorities. Green points are the predicted probability of STEM career interest for students who did not attend a STEM program. Blue points are the predicted probability of STEM career interest for students who attended a general population STEM program. Red points are the predicted probability of STEM career interest for students who attended a STEM program designed for underrepresented minorities.

Average adjusted predicted probabilities were calculated for model 2 (Fig 1). Red points in Fig 1 show the predicted probability that a student who attended a URM STEM program expressed interest in a STEM career at the end of high school. Likewise, blue points show the predicted probabilities for those who attended a general population STEM program, and green points are for those who did not attend any STEM program. Error bars represent one standard error of the predicted mean. For Black students who did not attend a STEM program, the predicted probability of STEM career interest was 27%. Black students who attended a general population STEM program gained a few percentage points (32%). We see that Black students who attended a URM STEM program had an impressive 42% probability of having a STEM career interest at the end of high school. The predicted probability for end of high school STEM career interest for Hispanic students who attended a URM program was 44%, compared to 33% for Hispanic students who attended a general population program and 29% for Hispanic students who did not attend a STEM program expressed an interest in a STEM career. For each racial and ethnic group, the red points in Fig 1 are significantly higher than the blue and green points. This indicates the positive benefit of URM STEM programs, compared with general population STEM programs.

## 5. Discussion

The systemic underrepresentation based on racial and ethnic demographics in the U.S. STEM labor force is a matter of substantial social injustice [51]. Underrepresentation exacerbates limitations on upward mobility and widens economic disparities between traditionally marginalized communities and traditionally dominant communities [52]. Additionally, a lack of racial and ethnic diversity in STEM is a hindrance to innovation and creativity [53]. By excluding certain groups, we risk missing out on valuable contributions that could advance scientific understanding and technological development. This work primarily aims to develop an understanding of factors that positively contribute to STEM career interest among URM high school students.

From our nationally representative sample of U.S. students, we found that students who attended a URM STEM program during high school had 2.4 times the odds of aspiring to a STEM career compared to their counterparts who did not engage in a STEM program. While studies of localized individual programs suffered from small sample size, findings from these programs are consistent with our results. For example, surveys from the RISE program showed that afterwards, 76% of students felt convinced a science career was right for them, and 81% felt better prepared and more motivated to pursue STEM careers [34]. Ninety-seven percent of RISE participants went on to attend 4-year institutions, 61% majored in STEM, and 20% conducted research [34]. Attendees of the Loma Linda summer research internship reported a 64-percentage point increase in their interest in scientific research-based careers from the pre-program survey to the post-program survey [26]. Follow up data from Loma Linda showed that 49% of program attendees were in college with 67% majoring in STEM and 55% enrolling in graduate programs [26]. The Loma Linda and RISE programs both provided students with financial support, connections to racially diverse mentors, career exposure, advanced STEM instruction, and assistance with college applications. These elements can be particularly beneficial for URM students who may have limited access to such resources in formal educational settings, where structural inequities—such as underfunded schools, underprepared teachers, and restricted access to advanced coursework [6,7,9]—can constrain students’ opportunities to develop STEM interests and skills.

We additionally assessed the relationship between students’ attendance of general population OST STEM programs and their subsequent reporting of STEM career interest. In comparison to those who did not attend any STEM programs, those who attended a general population program had 1.3 times the odds of expressing interest in a STEM career. This indicates that general population OST STEM programs are also associated with greater STEM career interest; however, the odds ratio is notably smaller than that observed for URM STEM programs. This finding is consistent with prior works that have used nationally representative data sets to assess the relationship between general population STEM program attendance and STEM career interest. For example, Kitchen et al. found that high school students who attended STEM summer programs had a 1.4 times increase in the odds of expressing a STEM career interest at the end of high school, compared to those who did not attend STEM summer programs [54]. Miller et al. found that students who participated in STEM competitions in high school had 1.2 times the odds of reporting interest in a STEM career at the end of high school compared with those who did not participate in competitions [55].

When directly comparing URM STEM programs to general population programs in model 3 (S1 Table), we found that those who attended a URM program as opposed to a general population program had 1.9 times the odds of expressing STEM career interest (p<0.001). This finding suggests that URM programs may be superior to general population STEM programs in promoting interest in a STEM career [22–26,28–31,33–35,37]. This result is in line with theoretical expectations from CRT. However, another remarkable finding was the absence of interactions between race, ethnicity, gender, first generation status, and STEM program type (S2-S4 Tables). Participation in URM OST STEM programs is associated with similarly higher STEM career interest across all participants in our U.S. college student sample. These results dovetail with the SCCT focus on non-race related factors. Some elements that are emphasized in the literature on URM STEM programs include connecting students to same race mentors and peers [22–29], incorporation of community oriented and culturally relevant STEM projects [29], financial support [26,28,31,34], and opportunities for parental engagement [29]. This raises an important question for follow-up research. What specific elements and pedagogical activities make the URM STEM programs so successful compared to general population programs? From an SCCT perspective, one might speculate that some of the above-mentioned strategies that URM STEM programs have implemented to support URM students are generally helpful for all students. Future research should identify which specific elements of URM-focused OST STEM programs are most impactful in order to inform the design and improvement of all OST STEM initiatives.

To our knowledge, this study is the first to explicitly report on the association between URM STEM programs and career interest using a nationally representative data set of students in the U.S. Prior studies have relied on small sample sizes from specific programs and have rarely controlled for prior interest or other confounding factors [22–26,28–31,33–35,37]. Our results offer more definitive and statistically robust evidence for the positive impact of URM STEM programs on cultivating interest in STEM careers.

## 6. Limitations

The statistical methods employed in this study are correlational and should not be interpreted as causal. Studies based on survey data may suffer from social desirability and recall biases [56]. Self-reported data introduce uncertainty about how students interpret survey questions. However, we employed appropriate psychometric methods to collect validity and reliability evidence for our instrument. This evidence supported the use of the survey. While we lack information on why students chose to attend a STEM program and their career interests immediately before attending, we do have beginning of high school STEM career interest as a control variable in our models as a proxy for pre-program interest. Given that early high school STEM career interest strongly predicts interest at the end of high school, we believe this serves as an adequate proxy. We acknowledge that our sampling method provided us with a nationally representative sample of college students in the U.S., and our results should not be generalized to other populations.

## 7. Conclusion

This work set out to answer our research question “Is URM STEM program attendance associated with increased STEM career interest?” Our findings suggest that STEM programs designed specifically for underrepresented minorities are a strong motivator for high school students’ STEM career aspirations. Programs such as these can involve engaging students in community oriented and culturally relevant STEM projects [29], connecting students with same race mentors and peers [22–29], providing students with financial support [26,28,31,34], and engaging parents in the program [22,29,33]. Given the nation’s growing population of people of color and the demand for STEM workers, URM STEM programs present a valuable opportunity that policymakers and funding agencies should prioritize. The findings from this study support continued and expanded funding for these programs, highlighting their role in shaping the STEM career paths of underrepresented minority students as well as in boosting STEM career interest among all students who participate in them.

## Supporting information

S1 File Supplementary Logistic Regression Models. This file includes four supplementary tables with additional logistic regression models.
(DOCX)

S2 File Survey Instrument. PDF of the complete survey instrument exactly as it appeared to students.
(PDF)

S3 File Raw Data. Spreadsheet containing all raw data necessary to replicate the results.
(XLSX)

S4 File R code for Step 1. R code used to perform multiple imputations.
(R)

S5 File R code for Step 2. R code used to perform multiple imputations.
(R)

S6 File R code for Step 3. R code used to perform multiple imputations.
(R)

S7 File R code for Step 4. R code used to create new variables.
(R)

S8 File R code for Step 5. R code used to calculate descriptive statistics.
(R)

S9 File R code for Step 6. R code used to generate logistic regression models and generate margins plot.
(R)
