# Supplementary material for: Increasing STEM career interest: The role of out-of-school time STEM programs designed for underrepresented minorities
Source: PLoS One. 2025 Nov 7;20(11):e0336418. doi: 10.1371/journal.pone.0336418 (PMC12594387; doi:10.1371/journal.pone.0336418)
Supplement: S1 File — This file includes four supplementary tables with additional logistic regression models. (DOCX) [file pone.0336418.s001.docx]

**S1 Table.** Logistic regression model predicting end of H.S. STEM career interest. This differs from model 2 solely by the reference level of the STEM program variable. Here, the reference is set as “general population STEM program” to allow for a direct comparison between a URM STEM program and a general population STEM program. Cells shaded grey are statistically significant at the 0.05 level or lower.

|  | **Model 3: Controls and Predictors** | | |
| --- | --- | --- | --- |
| **Variables** | **Odds Ratio (95% CI)** | **Standard Error** | **P-value** |
| Intercept | 0.30 (0.27-0.34) | 0.05 | <0.001 |
| **Controls** | | | |
| Beginning of H.S. STEM Career Interest | 8.01 (7.31, 8.80) | 0.05 | <0.001 |
| Black | 0.83 (0.69, 0.98) | 0.09 | 0.03 |
| Hispanic | 0.95 (0.84, 1.07) | 0.06 | 0.37 |
| Asian | 1.24 (1.08, 1.43) | 0.07 | 0.002 |
| Other race/eth | 1.02 (0.86, 1.22) | 0.09 | 0.80 |
| Female | 0.54 (0.49, 0.59) | 0.05 | <0.001 |
| First Generation | 0.99 (0.90, 1.10) | 0.05 | 0.88 |
| Math SAT | 1.32 (1.25, 1.40) | 0.03 | <0.001 |
| **Predictors** | | | |
| No program vs. General population STEM program | 0.77 (0.70, 0.84) | 0.05 | <0.001 |
| URM STEM program vs. General population STEM program | 1.85 (1.49, 2.29) | 0.11 | <0.001 |
| **Other model measures** | **AIC** | **BIC** | **N** |
|  | 13964 | 14047 | 14176 |

**S2 Table.** Logistic regression model predicting end of H.S. STEM career interest. This model includes an interaction between the race and ethnicity variable and the STEM program variable. For main effects, cells shaded grey are statistically significant at the 0.05 level or lower. For interaction terms, alpha was set to 0.001, thus none are statistically significant.

|  | **Model 4 race/eth*STEM program** | | |
| --- | --- | --- | --- |
| **Variables** | **Odds Ratio (95% CI)** | **Standard Error** | **P-value** |
| Intercept | 0.23 (0.20, 0.26) | 0.06 | <0.001 |
| **Controls** | | | |
| Beginning of H.S. STEM Career Interest | 8.02 (7.31, 8.81) | 0.05 | <0.001 |
| Black | 0.84 (0.64, 1.11) | 0.14 | 0.23 |
| Hispanic | 1.04 (0.86, 1.25) | 0.09 | 0.70 |
| Asian | 1.24 (0.98, 1.58) | 0.12 | 0.08 |
| Other race/eth | 1.06 (0.80, 1.41) | 0.15 | 0.68 |
| Female | 0.54 (0.49, 0.59) | 0.05 | <0.001 |
| First Generation | 0.99 (0.90, 1.10) | 0.05 | 0.88 |
| Math SAT | 1.32 (1.26, 1.40) | 0.03 | <0.001 |
| **Main effects** | | | |
| General population STEM program vs. No program | 1.35 (1.18, 1.54) | 0.07 | <0.001 |
| URM STEM program vs. No program | 3.91 (2.42, 6.31) | 0.24 | <0.001 |
| **Interaction terms** | | | |
| General population STEM program * Black | 1.03 (0.72, 1.47) | 0.18 | 0.89 |
| General population STEM program * Hispanic | 0.89 (0.70, 1.13) | 0.12 | 0.34 |
| General population STEM program * Asian | 0.99 (0.74, 1.32) | 0.15 | 0.93 |
| General population STEM program * Other race/eth | 0.98 (0.67, 1.42) | 0.19 | 0.90 |
| URM STEM program * Black | 0.53 (0.27, 1.04) | 0.35 | 0.07 |
| URM STEM program * Hispanic | 0.45 (0.24, 0.83) | 0.32 | 0.01 |
| URM STEM program * Asian | 0.86 (0.41, 1.80) | 0.37 | 0.70 |
| URM STEM program * Other race/eth | 0.47 (0.18, 1.20) | 0.48 | 0.11 |
| **Other model measures** | **AIC** | **BIC** | **N** |
|  | 13968 | 14112 | 14176 |

**S3 Table.** Logistic regression model predicting end of H.S. STEM career interest. This model includes an interaction between the female variable and the STEM program variable. For main effects, cells shaded grey are statistically significant at the 0.05 level or lower. For interaction terms, alpha was set to 0.001, thus none are statistically significant.

|  | **Model 5 female*STEM program** | | |
| --- | --- | --- | --- |
| **Variables** | **Odds Ratio (95% CI)** | **Standard Error** | **P-value** |
| Intercept | 0.23 (0.20, 0.25) | 0.06 | <0.001 |
| **Controls** | | | |
| Beginning of H.S. STEM Career Interest | 8.02 (7.30, 8.80) | 0.05 | <0.001 |
| Black | 0.83 (0.70, 0.98) | 0.09 | 0.03 |
| Hispanic | 0.95 (0.84, 1.07) | 0.06 | 0.38 |
| Asian | 1.25 (1.09, 1.43) | 0.07 | 0.002 |
| Other race/eth | 1.02 (0.86, 1.23) | 0.09 | 0.79 |
| Female | 0.57 (0.49, 0.66) | 0.07 | <0.001 |
| First Generation | 0.99 (0.90, 1.10) | 0.05 | 0.90 |
| Math SAT | 1.32 (1.25, 1.39) | 0.03 | <0.001 |
| **Main effects** | | | |
| General population STEM program vs. No program | 1.37 (1.21, 1.56) | 0.06 | <0.001 |
| URM STEM program vs. No program | 2.24 (1.55, 3.24) | 0.19 | <0.001 |
| **Interaction terms** | | | |
| General population STEM program * female | 0.90 (0.75, 1.08) | 0.09 | 0.27 |
| URM STEM program * female | 1.10 (0.70, 1.73) | 0.23 | 0.68 |
| **Other model measures** | **AIC** | **BIC** | **N** |
|  | 13966 | 14064 | 14176 |

**S4 Table.** Logistic regression model predicting end of H.S. STEM career interest. This model includes an interaction between the first-generation variable and the STEM program variable. For main effects, cells shaded grey are statistically significant at the 0.05 level or lower. For interaction terms, alpha was set to 0.001, thus none are statistically significant.

|  | **Model 6 first-generation*STEM program** | | |
| --- | --- | --- | --- |
| **Variables** | **Odds Ratio (95% CI)** | **Standard Error** | **P-value** |
| Intercept | 0.22 (0.19, 0.25) | 0.06 | <0.001 |
| **Controls** | | | |
| Beginning of H.S. STEM Career Interest | 8.02 (7.31, 8.80) | 0.05 | <0.001 |
| Black | 0.83 (0.69, 0.98) | 0.09 | 0.03 |
| Hispanic | 0.95 (0.84, 1.07) | 0.06 | 0.37 |
| Asian | 1.24 (1.08, 1.43) | 0.07 | 0.002 |
| Other race/eth | 1.02 (0.86, 1.22) | 0.09 | 0.81 |
| Female | 0.54 (0.49, 0.59) | 0.05 | <0.001 |
| First Generation | 1.11 (0.95, 1.29) | 0.08 | 0.17 |
| Math SAT | 1.32 (1.25, 1.39) | 0.03 | <0.001 |
| **Main effects** | | | |
| General population STEM program vs. No program | 1.42 (1.25, 1.61) | 0.06 | <0.001 |
| URM STEM program vs. No program | 2.72 (2.02, 3.66) | 0.15 | <0.001 |
| **Interaction terms** | | | |
| General population STEM program * first generation | 0.83 (0.69, 1.00) | 0.10 | 0.05 |
| URM STEM program * first generation | 0.77 (0.50, 1.20) | 0.23 | 0.25 |
| **Other model measures** | **AIC** | **BIC** | **N** |
|  | 13963 | 14061 | 14176 |
