# Supplementary material for: Increasing STEM career interest: The role of out-of-school time STEM programs designed for underrepresented minorities
Source: PLoS One. 2025 Nov 7;20(11):e0336418. doi: 10.1371/journal.pone.0336418 (PMC12594387; doi:10.1371/journal.pone.0336418)
Supplement: S2 File — PDF of the complete survey instrument exactly as it appeared to students. (PDF) [file pone.0336418.s002.pdf]

Harvard-Smithsonian Center for Astrophysics
Science Education Department

Survey of Students in Introductory College Classes

Researchers at the Harvard-Smithsonian Center for Astrophysics are interested in how your prior experiences, especially with out-of-school-time science activities, are shaping your career plans. By filling out this questionnaire you will help us find ways to improve science education for future students. Make your best estimate for each item and answer as many questions as possible. Your participation is, of course, voluntary. If you are under the age of 18, you may not participate in this study. Thank you for your help.

PROOF

This survey should take about 15–20 minutes to complete.

Confidentiality: This survey does not ask you for your name. Your anonymity is guaranteed. Any reports, published or unpublished, arising from this study, will include only averaged data of large groups.

Thank you for your time!

Use a No. 2 pencil or blue or black pen only.

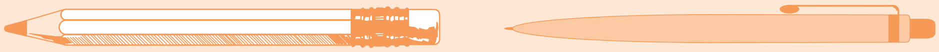

CORRECT MARK ⬬

INCORRECT MARKS ✓ ✗

This project is funded by the National Science Foundation, grant number 1612375: *How Pre-College Informal Activities Influence Female Participation in STEM Careers*. The lead researcher for this study is Dr. Philip M. Sadler, who can be reached at 617-496-4709 or by email at: psadler@cfa.harvard.edu.

DO NOT WRITE IN THIS AREA

SERIAL #

3/8" PERF

PROOF

3/8" PERF

NOTE: In this questionnaire, STEM stands for "Science, Technology, Engineering, Mathematics"

ABOUT YOUR CAREER PLAN DEVELOPMENT:

1. Which of the following describes what you want(ed) to be at the beginning of middle school, in high school (beginning and end), and at the beginning of college: *Mark all that apply. Leave blank those that do not apply.*

	Beginning of Middle School	Beginning of High School	End of High School	Beginning of first semester of College
Medical doctor (e.g., physician, dentist, vet.)	○	○	○	○
Health professional (e.g., social worker, nurse, pharmacist)	○	○	○	○
Astronomer	○	○	○	○
Biologist	○	○	○	○
Chemist	○	○	○	○
Earth/Environmental scientist	○	○	○	○
Physicist	○	○	○	○
Other scientist	○	○	○	○
Engineer	○	○	○	○
Computer scientist/Programmer/IT Specialist	○	○	○	○
Mathematician/Statistician	○	○	○	○
STEM teacher	○	○	○	○
Other teacher	○	○	○	○
Anthropologist/Archaeologist	○	○	○	○
Social scientist (e.g., psychologist, sociologist)	○	○	○	○
Humanities professional (e.g., historian, language specialist, writer, philosopher)	○	○	○	○
Visual artist (e.g., painter, sculptor, architect, computer artist/ animator)	○	○	○	○
Performing artist (e.g., actor, musician, dancer)	○	○	○	○
Business person (e.g., entrepreneur, manager)	○	○	○	○
Lawyer	○	○	○	○
Politician	○	○	○	○
Athlete/Coach	○	○	○	○
Military personnel	○	○	○	○
Other non-STEM related career	○	○	○	○

PROOF

2. Rate the following factors in terms of their importance for your future career satisfaction:

Not at all important	0	1	2	3	4	5	Extremely important
Making money	0	1	2	3	4	5	
Becoming well known	0	1	2	3	4	5	
Helping other people	0	1	2	3	4	5	
Having others working under my supervision	0	1	2	3	4	5	
Having job security	0	1	2	3	4	5	
Working with people rather than objects	0	1	2	3	4	5	
Inventing new things	0	1	2	3	4	5	
Developing new knowledge and skills	0	1	2	3	4	5	
Having lots of family time	0	1	2	3	4	5	
Having lots of time for myself/friends	0	1	2	3	4	5	
Making my own decisions	0	1	2	3	4	5	
Having an easy job	0	1	2	3	4	5	
Having an exciting job	0	1	2	3	4	5	
Making use of my talents/abilities	0	1	2	3	4	5	
Working in an area with lots of job opportunities	0	1	2	3	4	5	
Having a creative job	0	1	2	3	4	5	

ABOUT YOUR EARLY SCHOOL SCIENCE AND MATH EXPERIENCES:

3. How would you rate your science experiences in elementary grades (K-4)?

Very memorable in a negative way	0	1	2	3	4	5	Very memorable in a positive way
	0	1	2	3	4	5	

4. What was your average grade in middle school (5-8)? *Letter grades include intermediate grades (e.g., A-, C+).*

	A	B	C	D	F
English/Language Arts	A	B	C	D	F
Mathematics	A	B	C	D	F
Science	A	B	C	D	F

NEXT →

DO NOT WRITE IN THIS AREA

SERIAL #

3/8" PERF

NOTE: In this questionnaire, STEM stands for "Science, Technology, Engineering, Mathematics"

5. At the end of middle school, how interested were you in:

	Not at all interested 0	1	2	3	4	5 Extremely interested
Science	0	1	2	3	4	5
Mathematics	0	1	2	3	4	5
Engineering	0	1	2	3	4	5
Computing	0	1	2	3	4	5

ABOUT YOUR HIGH SCHOOL BACKGROUND:

6. What type of school did you go to? *Mark all that apply.*

- Public
- Private/Independent
- Public Charter
- Private Religious
- Magnet School
- Vocational
- International Baccalaureate
- Single-sex
- Outside of US
- Home Schooled

7. To help us estimate the size of the community you come from, please provide your home ZIP Code when you went to high school and bubble in the corresponding numbers.

ZIP CODE				
0	0	0	0	0
1	1	1	1	1
2	2	2	2	2
3	3	3	3	3
4	4	4	4	4
5	5	5	5	5
6	6	6	6	6
7	7	7	7	7
8	8	8	8	8
9	9	9	9	9

8. What grade did you get in your last high school English course? *Letter grades include intermediate grades (e.g., A-, C+).*

- A
- B
- C
- D
- F

9. Which of the following mathematics courses did you take in high school? *Mark all that apply.*

- Trig./Analytic Geometry
- Statistics
- Pre-Calculus
- AP Statistics
- Calculus
- Integrated Math
- AP Calculus AB
- AP Calculus BC

PROOF

10. For the most advanced mathematics course you took, what was your final grade? *Letter grades include intermediate grades (e.g., A-, C+).*

- A
- B
- C
- D
- F

11. For each of the following standardized tests, please indicate the score you earned on each subtest taken by marking the appropriate numbers.

SAT Score	SAT Exam-pre March 2016: Math Subtest	SAT Exam-pre March 2016: Writing Subtest	SAT Exam-pre March 2016: Critical Reading Subtest	New SAT: Reading and Writing Subtest	New SAT: Math Subtest	ACT Score	ACT Exam: Math Subtest	ACT Exam: English Subtest	ACT Exam: Science Subtest	ACT Exam: Reading Subtest
200–300						1–11				
310–400						12–15				
410–500						16–20				
510–600						21–25				
610–700						26–30				
710–800						31–36				

12. For the following high school STEM courses you took, please indicate the level of the course. *Mark only ONE course level per row.*

HS Course Subject	Course Level: Regular	Honors	AP	IB	Dual enrollment
Phyiscal Science					
1st Biology course					
2nd Biology course					
1st Chemistry course					
2nd Chemistry course					
1st Physics course					
2nd Physics course					
Engineering					
Computer Science					

3/8" PERF

NOTE: In this questionnaire, STEM stands for "Science, Technology, Engineering, Mathematics"

13. At the end of high school, how interested were you in:

	Not at all interested 0	1	2	3	4	5 Extremely interested
Science	0	1	2	3	4	5
Mathematics	0	1	2	3	4	5
Engineering	0	1	2	3	4	5
Computing	0	1	2	3	4	5

ABOUT YOUR STEM-RELATED INTERESTS:

14. Which of the following experiences did you have while growing up? *Mark all that apply. Leave blank those that you did not have.*

	If you had these experiences, please mark how often and during which grades you participated					
	K-4		5-8		9-12	
	Sometimes	Often	Sometimes	Often	Sometimes	Often
Using tools to tinker with/take apart **mechanical** devices (e.g., bicycle, watch, door lock)						
Using tools to tinker with/take apart **electrical** devices (e.g., hair dryer, hand mixer, TV, computer)						
Baking/cooking/kitchen chemistry						
Using science equipment (e.g., microscope, telescope)						
Using STEM toys/kits (e.g., building/construction sets, circuit boards, model rockets, science kits)						
Playing strategy board games/logic games or puzzles						
	Sometimes	Often	Sometimes	Often	Sometimes	Often
Reading **non-fiction** science (e.g., news, books, magazines, journals - hardcopy or online)						
Reading science **fiction** (hardcopy or online)						
Watching STEM-related TV programs or movies (documentaries, dramas, sci-fi.)						
Watching online STEM-related videos (e.g., YouTube)						
Playing STEM computer/video games						
Following STEM on social media						
Using STEM apps (eg., SkyView, BrainPOP, Touch Surgery)						
Writing about STEM, including creating online blogs/podcasts/videos						
Writing computer programs/games/apps or designing web pages						
	Sometimes	Often	Sometimes	Often	Sometimes	Often
Taking care of/raising/training an animal						
Indoor/outdoor gardening						
Observing/documenting animals (e.g., bird-watching)						
Collecting things in nature (e.g., rocks, seashells)						
Observing or studying stars and other astronomical objects						
Observing clouds or weather patterns						
Taking/editing photographs or videos of nature						
Exploring nature while walking/hiking/ camping/snorkeling/ geocaching, etc.						
Collecting/analyzing data for scientists (online, with apps, or in person, e.g., Zooniverse)						

PROOF

NEXT

3/8" PERF

NOTE: In this questionnaire, STEM stands for "Science, Technology, Engineering, Mathematics"

15. Did you participate in any of the following STEM programs/activities during your middle or high school years?
Mark all that apply. Leave blank those that do not apply.

	If you participated in any of these activities, please mark how often and in which grades you participated				This activity increased my interest in STEM
	5-8		9-12		
	Sometimes	Often	Sometimes	Often	Mark if yes
STEM-related extracurricular clubs/teams at school					
STEM-related clubs/teams outside of school					
Group organization (e.g., Girl Scouts, Boy Scouts, 4H)					
Maker/DIY STEM activities/events					
Overnight STEM programs (at museums, science centers, etc.)					
STEM Cafes (eat, drink, chat about STEM with professionals)					
STEM-related vacation or summer camps					
STEM-related programs that collect/analyze data for scientists (e.g., citizen science)					
	Sometimes	Often	Sometimes	Often	Mark if yes
STEM-related lectures or talks (online or in person)					
STEM-related courses/workshops outside of school (online or in person)					
STEM-related leadership conferences					
Science fairs					
Robotics competitions					
Engineering competitions					
Computing/IT competitions					
STEM-related academic/research summer programs					
	Sometimes	Often	Sometimes	Often	Mark if yes
STEM-related career days					
Tours of STEM-related settings (e.g., hospital, vet's office, lab)					
STEM-related job-shadowing					
STEM-related internships					
Work/Volunteer in a STEM-related setting (e.g., hospital, vet's office, lab, camp, museum, zoo)					

PROOF

16. If you participated in STEM programs/activities <u>outside of school</u>, what was your experience with the following opportunities:

	I experienced this STEM opportunity	This opportunity increased my interest in STEM	This opportunity showed the real-life relevance of STEM
	Mark if yes	Mark if yes	Mark if yes
Interacting with a STEM mentor			
Interacting with a STEM role model			
Interacting with someone who works in a STEM career			
Working with older STEM students (college students)			
Taking on a leadership role			
Mentoring/tutoring younger students in STEM			
	Mark if yes	Mark if yes	Mark if yes
Participating in hands-on STEM activities			
Participating in programs with art and/or design connections (STEAM)			
Working on real world STEM issues/problems			
Using STEM equipment to collect data			
Building/constructing STEM models			
Designing and carrying out my own STEM project			
Working with others on a team			
Presenting STEM data/information to others (e.g., poster, paper, oral presentation)			
Learning about STEM careers			

3/8" PERF

DO NOT WRITE IN THIS AREA

SERIAL #

NOTE: In this questionnaire, STEM stands for "Science, Technology, Engineering, Mathematics"

17. Did you attend any single sex STEM programs?

- yes
- no

18. Did you attend any STEM programs designed specifically for underrepresented minorities?

- yes
- no

19. If you had someone you consider a STEM role model, were they reflective of your ***(mark all that apply)*****:**

- gender identity
- ethnic identity
- racial identity
- not reflective of my gender, ethnic , or racial identity

20. If you did NOT attend any STEM programs/activities outside of school, please indicate why. ***Mark all that apply.***

- I didn't know STEM opportunities were available in my area
- I looked, but there were no STEM opportunities available in my area
- STEM opportunities were available but I didn't have the time (other commitments: work/home/other activities) to attend
- STEM opportunities were available but I didn't have the resources (transportation/finances) to attend
- STEM opportunities were available but I was not interested in the specific topics offered
- STEM opportunities were available but I was not interested in STEM
- STEM opportunities were available but I didn't feel welcome/comfortable attending
- Other: ______________________

21. Some people say their interest (or lack of interest) in STEM is the result of many events. Others say there was one defining event (seeing something, hearing something, learning something, meeting someone, etc.). Which best describes your experiences?

- Many events
- One single event

If a single event, when did it occur: K–4 / 5–8 / 9–12

If a single event, where did it occur: in school / outside of school

PROOF

22. To what extent do you disagree or agree with the following statements:

No, Not at all	0	1	2	3	4	5	Yes, Very much
Topics in STEM excite my curiosity	0	1	2	3	4	5	
I enjoy learning about STEM	0	1	2	3	4	5	
I like to know what is going on in STEM	0	1	2	3	4	5	
I feel confident in my ability to learn STEM	0	1	2	3	4	5	
Others ask me for help in STEM	0	1	2	3	4	5	
I can do well on tests/exams in STEM	0	1	2	3	4	5	
I understand concepts I have studied in STEM	0	1	2	3	4	5	
I can overcome setbacks in learning STEM	0	1	2	3	4	5	
I am interested in learning more about STEM	0	1	2	3	4	5	
I see myself as a STEM person	0	1	2	3	4	5	
My family sees me as a STEM person	0	1	2	3	4	5	
My friends/classmates see me as a STEM person	0	1	2	3	4	5	
My classroom STEM teachers see me as a STEM person	0	1	2	3	4	5	
My out-of-school teachers see me as a STEM person	0	1	2	3	4	5	
I am aware of many types of STEM-related careers	0	1	2	3	4	5	
I am aware of the skills STEM professionals use	0	1	2	3	4	5	
I feel I belong in the STEM community	0	1	2	3	4	5	

ABOUT YOURSELF AND YOUR FAMILY:

23. Gender? Male / Female / Other: ______________________

24. Are you of Hispanic origin? yes / no

25. What is your race? ***(For multi-racial, mark all that apply.)***

- Black
- White
- Asian or Pacific Islander
- American Indian or Alaskan Native
- Other: ______________________

26. Was English the primary spoken language in your household? yes / no

27. What year are you in college? Freshman / Sophomore / Other: ______________________

NEXT →

3/8" PERF

NOTE: In this questionnaire, STEM stands for "Science, Technology, Engineering, Mathematics"

28. What was the highest level of education for your parents/guardians?

	Male	Female	Less than High School Diploma	High School Diploma/GED	Some College/ Associate Degree	Bachelor's Degree	Master's Degree or higher	Not applicable
Parent/guardian #1	○	○	○	○	○	○	○	○
Parent/guardian #2	○	○	○	○	○	○	○	○

29. Which category best fits you and your parents' or guardians' background?

	Born in United States		
You	○ Yes	○ No	
Parent or Guardian #1	○ Yes	○ No	○ Not applicable or Don't know
Parent or Guardian #2	○ Yes	○ No	○ Not applicable or Don't know

30. Which of the following statements describes your family's interest in, and attitudes toward STEM? ***Mark all that apply.***

- ○ STEM is involved in career of parent #1.
- ○ STEM is involved in a sibling's career.
- ○ STEM is considered a diversion or hobby.
- ○ STEM is not a family interest.
- ○ Someone could help me with STEM homework.
- ○ My family arranged my attendance in out of school STEM programs.
- ○ STEM is involved in career of parent #2.
- ○ STEM is involved in another relative's career.
- ○ STEM is a way for people to have a better career.
- ○ STEM was a series of courses that I had to pass.
- ○ My family supported my attending STEM programs/activities.

PROOF

31. How often have you done the following?

	With family				With friends			
	Not at all			Very often	Not at all			Very often
Talk about STEM	○	○	○	○	○	○	○	○
Participate in STEM activities	○	○	○	○	○	○	○	○
Visit science centers, museums, nature centers, etc.	○	○	○	○	○	○	○	○
Attend STEM event/programs	○	○	○	○	○	○	○	○

32. Who encouraged you to select a *STEM* career path? ***Mark all that apply.***

- ○ No One
- ○ Sibling
- ○ Elementary School Teacher
- ○ School Counselor
- ○ Other:
- ○ Parent/Guardian #1
- ○ Other relative
- ○ Middle School Teacher
- ○ Out-of-School Teacher
- ○ Parent/Guardian #2
- ○ Friend
- ○ High School Teacher
- ○ Mentor or Role Model

→ ______________________________

33. Who encouraged you to select a *non-STEM* career path? ***Mark all that apply.***

- ○ No One
- ○ Sibling
- ○ Elementary School Teacher
- ○ School Counselor
- ○ Other:
- ○ Parent/Guardian #1
- ○ Other relative
- ○ Middle School Teacher
- ○ Out-of-School Teacher
- ○ Parent/Guardian #2
- ○ Friend
- ○ High School Teacher
- ○ Mentor or Role Model

→ ______________________________

You have reached the end of the survey. Thank you very much for your time!

SCANTRON Mark Reflex® EM-300746-1:654321

DO NOT WRITE IN THIS AREA

SERIAL #

3/8" PERF
